# Pairwise Heuristic Sequence Alignment Algorithm Based on Deep Reinforcement Learning

**DOI:** 10.1109/OJEMB.2021.3055424

**Published:** 2021-01-29

**Authors:** Yong-Joon Song, Dong Jin Ji, Hyein Seo, Gyu Bum Han, Dong-Ho Cho

**Affiliations:** School of Electrical EngineeringKorea Advanced Institute of Science and Technology 34968 Daejeon 305-701 South Korea

**Keywords:** Deep reinforcement learning, global alignment, pairwise alignment, sequence alignment, sequence comparison

## Abstract

*Goal:* Various methods have been developed to analyze the association between organisms and their genomic sequences. Among them, sequence alignment is the most frequently used method for comparative analysis of biological genomes. We intend to propose a novel pairwise sequence alignment method using deep reinforcement learning to break out the old pairwise alignment algorithms. *Methods:* We defined the environment and agent to enable reinforcement learning in the sequence alignment system. This novel method, named DQNalign, can immediately determine the next direction by observing the subsequences within the moving window. *Results:* DQNalign shows superiority in the dissimilar sequence pairs that have low identity values. And theoretically, we confirm that DQNalign has a low dimension for the sequence length in view of the complexity. *Conclusions:* This research shows the application method of deep reinforcement learning to the sequence alignment system and how deep reinforcement learning can improve the conventional sequence alignment method.

## I. Introduction

Recent advancements in sequencing technology have enabled the analysis of organisms with long sequences [1]. In case of organisms with short sequences, the evolutionary distances between the organisms could be easily analyzed using the older pairwise alignment methods, such as the conventional Needleman–Wunsch (NW) algorithm [2], and the relationship between the organisms could be investigated. However, the conventional pairwise alignment method is extremely simple to determine the characteristics of genomic sequences; therefore, it is difficult to balance the complexity and performance. Various attempts have failed in long sequence alignment. The conventional dynamic programming based NW alignment method has a complexity that is proportional to the product of the lengths of the two different nucleotide sequences, which causes great difficulty from the alignment perspective.

The sequence alignment method has reduced the complexity through the development of a multiple sequence alignment (MSA) method that aligns multiple sequences simultaneously. This method has been improved through various approaches, such as detecting common subsequences, ordering the progressive alignment, or increasing the speed using multithreads based on GPUs [3]–[5]. However, there remains a critical point in that the complexity of pairwise alignment could be more severe in case of MSA [6].

In particular, several pairwise alignment algorithms, such as banded alignment, the BLAST, and the MUMmer have been proposed to improve the speed of pairwise alignment [7]–[9]. These alignment methods attempted to solve the complexity issue by limiting the range of the alignment and extending the alignment after word matching or average common substring matching. However, in these cases, there were some accuracy problems in the process of extending short local alignments to large and complex sequences.

To overcome the problems of conventional alignment methods, we proposed a novel alignment method using deep reinforcement learning agent. Reinforcement learning is a way to teach an agent that could choose the best actions by observing an environment in a given system. The conventional tabular based reinforcement learning has a difficulty in expiring and learning the massive and complex systems. To improve this, the deep reinforcement learning method was proposed, and it overcame the limitations by approximately learning the complex systems [10]. The development of reinforcement learning has shown amazing performance in various complex systems [11]–[13]. Therefore, we decided to apply this deep reinforcement learning method to sequence alignment system that is willing to find the optimal matches in two complete sequences. Thus, we will describe the application of the deep reinforcement learning to the sequence alignment system in this paper.

The key contribution of this paper is as follows:

-- Apply the deep reinforcement learning algorithm to the sequence alignment system
-- Investigate the effect of each parameter on the performance of sequence alignment
-- Verify alignment performance superiority in short fairly dissimilar sequence pairs
-- Combine the proposed DQNalign with the conventional sequence alignment algorithm
-- Prove how the proposed DQNalign can obtain the optimal alignment's performance

## II. Materials and Methods

**Fig. 1.**
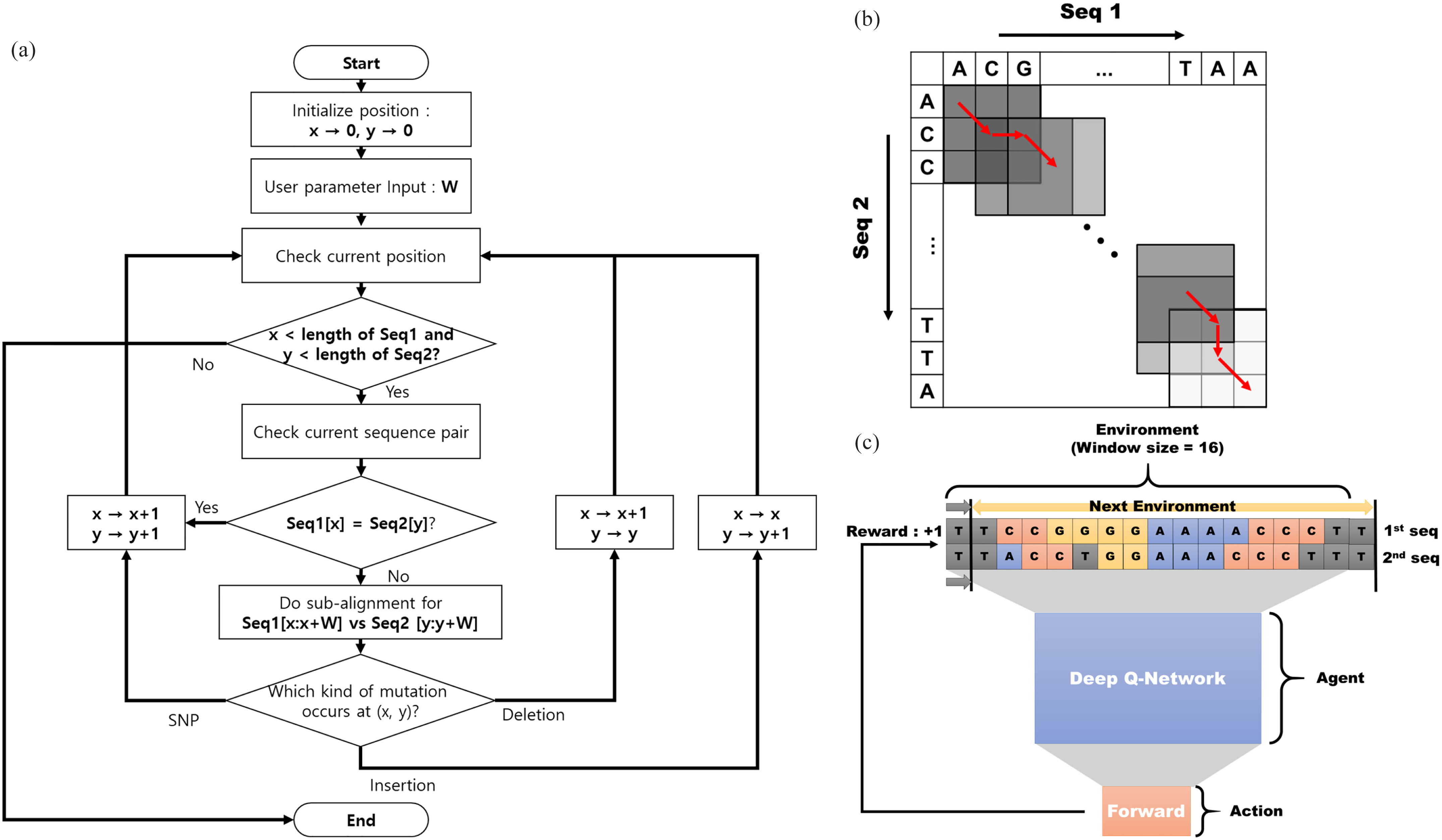
(a) Total process of the proposed DQNalign method. DQNalign is divided into two parts. First, (b) Local best path selection method, which is a method of repeating window movement by setting direction of progress through alignment process between small sub-sequences. Second, c) deep reinforcement learning-based local best path selection method, which solves high complexity problem of repeating small alignments. Reinforcement learning defines subsequences of current window as environment, and agent is defined as deep reinforcement learning. Here, reinforcement learning proceeds based on rewarding according to scoring strategy of sequence alignment.

Through this paper, we will introduce the pairwise heuristic sequence alignment algorithm based on deep reinforcement learning. We call this proposed method as DQNalign. The entire procedure of DQNalign is described as the flow chart in Fig. 1(a). The detailed explanation and the code implementation of DQNalign algorithm are available at https://github.com/syjqkrtk/DQNalign. Here, the proposed DQNalign algorithm can include one deep neural network structure among the two types that are used to predict the processing direction of alignment efficiently: Dueling Double Deep Q-network (DDDQN), faster Dueling Double Deep Q-network (faster DDDQN). The detailed network architecture will be explained in this section.

### A. Deep Reinforcement Learning in Sequence Alignment System

To apply deep reinforcement learning to sequence alignment, we try to develop a novel sequence alignment method using reinforcement learning. Instead of observing the entire sequence at once, we propose a novel heuristic sequence alignment method that repeats the small alignment while moving the window of sub-sequence pairs. Through DQNalign, it is possible to solve memory and time complexity problems.

The proposed heuristic sequence alignment method can be expressed as shown in Fig. 1(b), and we call this proposed heuristic alignment method as local best path selection method. The problem of determining the optimal direction in sub-sequence within a window can also be seen as a kind of sub-alignment process. It can also be inferred that if the window size is expanded to the entire sequence length, the local best path selection method turns into the optimal sequence alignment. We prove this relation between window size and performance through a numerical analysis, which will be discussed in the result section. Also, we propose a novel deep reinforcement learning based sequence alignment system shown in Fig. 1(c). After 2 sub-sequences in the window are set as environment, the agent with the deep Q-network observes the current environment and selects the direction (forward, insertion, deletion) as the next action. The scoring system of conventional alignment method will be used as reward in reinforcement learning. Through this process, we can execute deep reinforcement learning-based agent that can find the optimal path of the alignment at a given position.

### B. Network Architecture

In order to apply sequence alignment based on deep reinforcement learning, we have defined and used two network architectures: 1) Dueling Double Deep Q-network (DDDQN) and 2) Faster Dueling Double Deep Q-network: Separable convolutional layer based acceleration (faster DDDQN). We considered two types: DDDQN for high accuracy and faster DDDQN for 2x speed, and we used multiple cases of these networks by changing windows size. Detailed description of each network architecture is given in Supplementary materials S1, and their structures are given in Fig. S1 and Fig. S2.

### C. Training Procedure

Most machine learning requires a massive number of input data. In addition, using only a limited set of data in particular sequences can cause bias in deep reinforcement learning. Therefore, we created new training environments according to the model of evolution.

In the training procedure, we generated the sequence pairs according to following rules. First, we generated a completely random sequence. Then, we made the other sequence by mutating the random sequence. For convenience, we used the JC69 model to create the SNP mutations [19]. We used the Zipfian distribution based indel length model for generating the indels [20].

### D. Conventional Sequence Alignment Algorithms

In the DQNalign method, the starting point can critically affect the result because the alignment procedure is performed only in one direction. Therefore, we improved stability by applying the preprocessing methods of conventional alignment methods. We applied the longest common substring method to HEV, *E.coli* and mammalian cases, and the MUMmer method was used only in the *E.coli* case. Then, we will briefly explain how these conventional methods are adopted to the proposed DQNalign method.

#### 1) Longest Common Substring

We extracted the longest common substring using SequenceMatcher function in difflib of Python. Using this function, the most similar portion in the sequence pair was found. Then, this longest common substring was used as a starting point of the DQNalign method.

#### 2) Mummer

For the purpose of using the MUMmer software as the preprocessing of our DQNalign method, we used the official version of the MUMmer software in https://sourceforge.net/projects/mummer/. MUMmer can obtain the entire global alignment by itself, but dissimilar parts of the sequence pair could be skipped. To improve the coverage of the MUMmer, we apply our DQNalign method to process these ignored parts. For these ignored parts, the DQNalign algorithm aligns until it meets the aligned elements.

## III. Results

To show the feasibility and performance of the overall proposed DQNalign algorithms, we designed the following three simulations: 1) Numerical analysis on the step error probability, 2) Performance difference among DQNalign methods according to parameter changes, and 3) Performance comparison with the conventional alignment methods. Based on these simulations, we will show how the DQNalign method can improve the heuristic sequence alignment performance and how we can adapt the DQNalign method into the conventional alignment methods.

### A. Numerical Analysis and Results

Before applying the deep reinforcement learning, it is necessary to prove clearly whether the heuristic sequence alignment method in Fig. 1 can perform properly. In previous researches,the distribution of the alignment score has been expressed by various distribution formulas such as Gumbel distribution and gamma distribution [14]–[16]. In this paper, we intend to derive the step error probability using the alignment score distribution based on the Gumbel distribution because [14] derived the relationship between sequence lengths and alignment score. Thus, we cited the following equation for Gumbel distribution as the score distribution of the alignment in section 4 of [14].

\begin{equation*}
P(S(m,n) \leq s) \sim \exp (-Kmne^{-\lambda s}) \tag{1}
\end{equation*}

Here, $S(m,n)$ is the distribution of alignment scores between two sequences with lengths $m$ and $n$. $K$ and $\lambda$ are constants determined by the alignment environment. Using this equation, we proved that the local best path selection method can perform an optimal alignment in case of large window sizes.

#### 1) Numerical Analysis on the Step Error Probability

In order to approximate the step error probability in a given environment, we have defined the following three major assumptions and one constraint. Then, we can infer a rough relationship between the window size and the step error probability. First, we assume that the NW algorithm can precisely match the mutation information of the actual sequences. Second, it is assumed that several alignment paths are well-aligned when they have the same highest score in the current window. Third, the alignment at each step in the local best path selection method can be calculated independently. Conversely, for the constraint, we consider the alignment scoring parameters to prevent the indel preferences. We can derive equations on the error probability in case of similar sequences that have positive alignment scores.

**Fig. 2.**
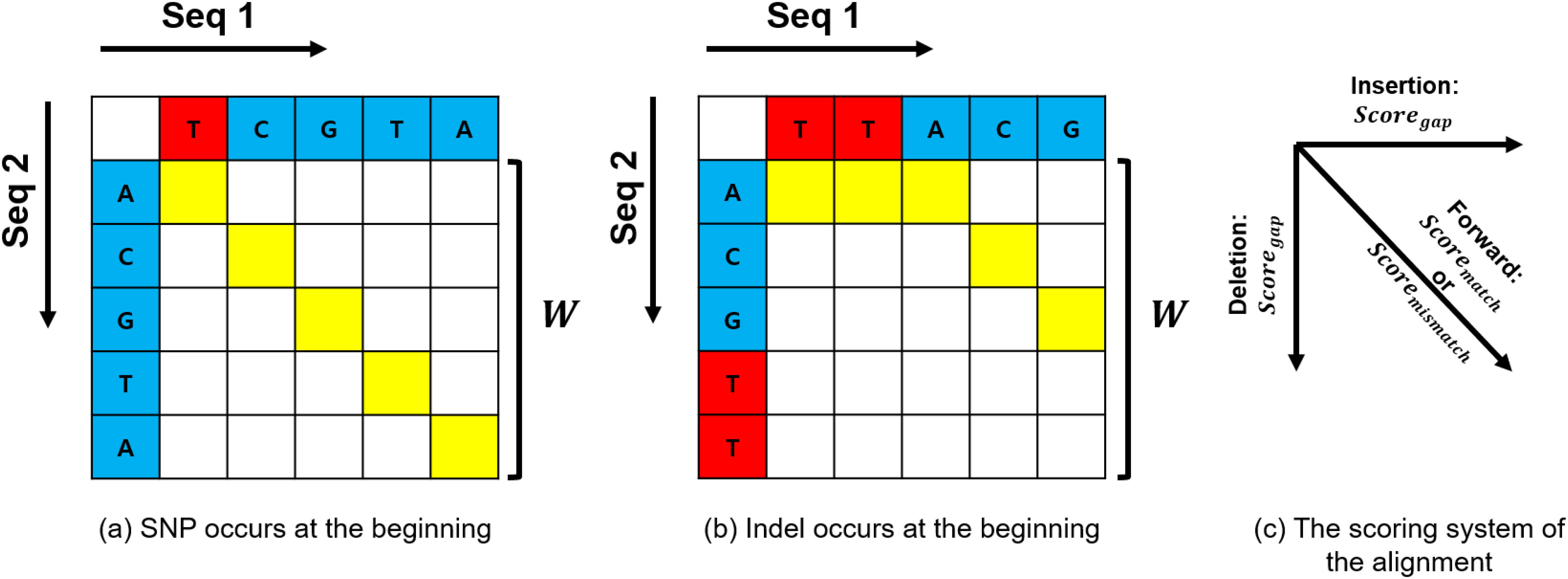
Modeling of local best path selection model for analysis of error probability in case of a fixed window size. (a) Expression of SNP within window size. (b) Expression of an indel within window size. (c) The scoring system of the alignment.

Here, we calculate the step error probability of the local best path selection method by considering the SNP and the indel cases seperately. The case of the SNP occurrence is depicted in Fig. 2(a). If the score of the optimal alignment is $score_{ans}$, we can say that an error occurs when the score of indel direction is higher than $score_{ans}$. In addition, $W$ means the window size, and $score_{gap}$ means the gap penalty of the alignment system. Then, the step error probability can be expressed using the Gumbel distribution as follows.

\begin{align*}
&P(S(W,W-1) + {score}_{gap} > {score}_{ans})\\
&\simeq 1 - \exp (-KW(W-1)e^{-\lambda ({score}_{ans}-{score}_{gap})}) \tag{2}
\end{align*}

Here, the $score_{ans}$ can be expressed as $Wscore_{avg}$. Then, the step error probability can be summarized in case of infinitely large W as following.

\begin{equation*}
\begin{aligned} P_{e,SNP} \simeq \lim _{W \to \infty } 2Ke^{\lambda {score}_{gap}} \frac{W^{2}}{e^{\lambda W {score}_{avg}}} \to 0 \end{aligned} \tag{3}
\end{equation*}

We confirm that the error probability, $P_{e,SNP}$, converges to 0 when W is infinitely large. The detailed derived process is summarized in supplementary material S1. Based on the process used to derive the step error probability in case that SNP occurs, we can analyze the step error probability, $P_{e,indel}$, for the indel environment in Fig. 2(b), which is expressed as follows.

\begin{align*}
P_{e,indel} \simeq &K \left(e^{\lambda {score}_{gap}}\!+\!\frac{1}{4}e^{\lambda {score}_{match}}\!+\!\frac{3}{4}e^{\lambda {score}_{mismatch}}\right)\\
&\times \lim _{W \to \infty } \frac{W^{2}}{e^{\lambda W {score}_{avg}}} \to 0 \tag{4}
\end{align*}

Here, $score_{match}$ means the match score, and $score_{mismatch}$ means mismatch score in the alignment system. Finally, the total step error probability, $P_{total}$, including the occurrence probability of indel, $P_{indel}$ and that of SNP, $P_{SNP}$ is as follows.

\begin{equation*}
\begin{aligned}[b]
P_{e,total} \simeq &\left({p}_{indel} K\left(e^{\lambda {score}_{gap}}+\frac{1}{4}e^{\lambda {score}_{match}}\right.\right.\\
& +\left.\frac{3}{4}e^{\lambda {score}_{mismatch}}\right)\\
&+\left. 2{p}_{SNP}Ke^{\lambda {score}_{gap}}\vphantom{\frac{1}{4}e^{\lambda {score}_{match}}}\right) \frac{W^{2}}{e^{\lambda W {score}_{avg}}} \to 0 \end{aligned} \tag{5}
\end{equation*}

#### 2) Numerical Results for the Step Error Probability Analysis

To verify the results of this analysis, we generated in-silico sequences according to the model of evolution. Next, we compared the results of alignment in the total length with that in a fixed window size. Under the second assumption, all of the paths with the same score were treated as correct answers. The detailed simulation scenario is described in Table S2. In Fig. S3, we confirmed that the simulation result exhibits a similar tendency compared to the analysis results based on Eq.(5). As expected, we observed that the error rate is close to zero when the window size is large.

### B. Simulation Results for the Various Types of DQNalign Method

We designed the following two simulations to analyze the performance according to various parameters in the proposed scheme: 1) performance convergence of the deep reinforcement learning-based alignment in the training procedure and 2) performance analysis according to various parameters. Through these simulations, we showed the processes adapting deep reinforcement learning to alignment and considering the optimal parameters of the DQNalign method.

#### 1) Performance Convergence in the Training Procedure

**Fig. 3.**
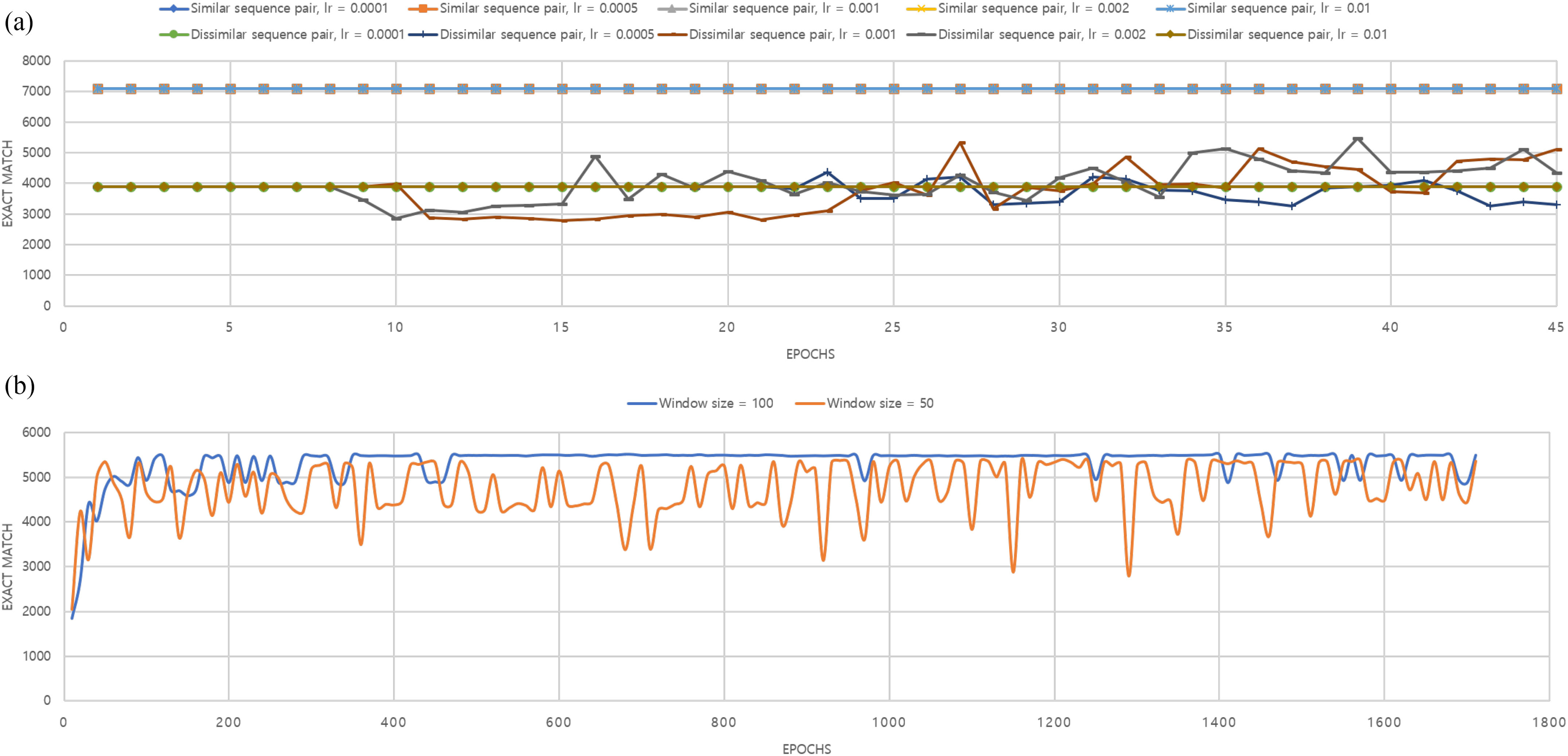
Performance convergence graph of DQNalign method. (a) The tendency changes of the convergence according to various learning rates for faster DDDQN in case that the window size is 100. (b) The convergence behavior of faster DDDQN in total training procedure in case of dissimilar sequence pair

The performance convergence of DQNalign method in training procedure is shown in Fig. 3. We prepared two HEV genome sequence pairs to evaluate the training process. One was a similar sequence pair (B1(Bur-82) vs. B2(Bur-86)) and the other was a dissimilar sequence pair (B1(Bur-82) vs. HE-JA1).

In case of similar sequence pair, we could see convergence within a short time regardless of the learning rates in the training procedure. This phenomenon occurs because the environment has rare indel cases in the training procedure. Therefore, the AI learns the wrong behavior that obtains high scores by judging only the forward direction regardless of the environment.

However, it is necessary to learn various environments while evading the biased results to align the actual sequence pairs. Fig. 3(a) shows the test results in a challenging environment in case of dissimilar sequence pairs. As can be seen in the result, the agent could not learn the alignment process in case of improper learning rates, and it can escape the biased results in case of proper learning rates. Therefore, we could figure out that it is crucial to tune the learning rates and the other parameters. Then, we could get the final convergence graph in Fig. 3(b). Here, by adjusting the learning rates, we could see that the network converge within thousands of epochs. We used these converged networks to evaluate the performance of DQNalign method. Also, you can download the converged network from our Github link.

#### 2) Effect of Parameters

**Fig. 4.**
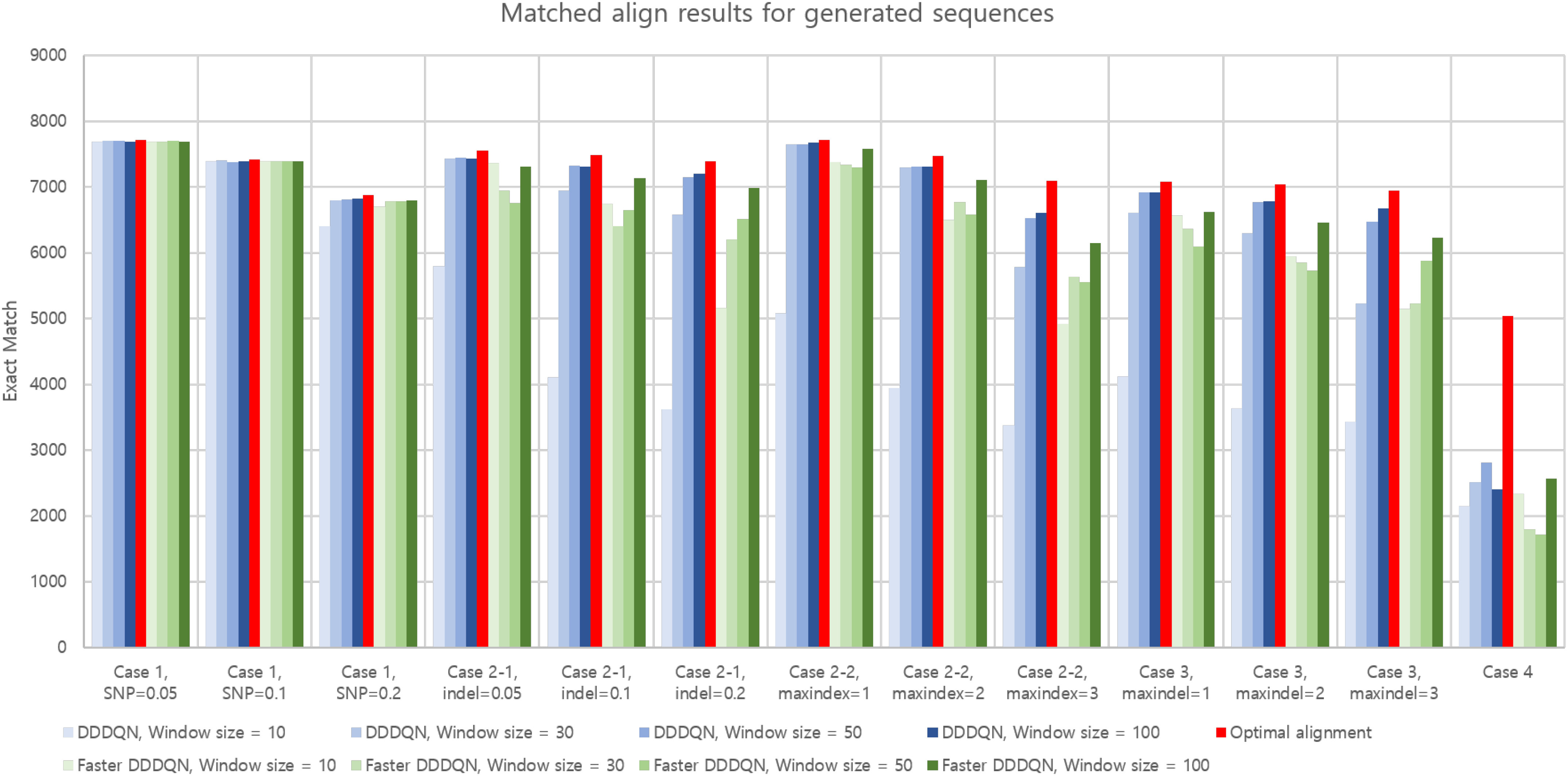
Exact match results of DQNalign method and optimum results in various simulation scenarios.

To observe the effect of the parameters, we used the simulation environments in Table S3. Here, we compared the exact match result of DQNalign method with that of conventional alignment methods. Also, we considered the Needleman–Wunsch algorithm's result to know the ideal outcome of each simulation case. Then, the result of sequence alignment in the generated sequences for the simulation cases in Table S3 is shown in Fig. 4.

As shown in Fig. 4, an increase in the window size increased the alignment performance. When the window size is 100, the performance was converged. Therefore, we could see that 100 is the optimal window size for these simulation cases.

Moreover, DQNalign method is not limited to a specific neural network structure, which means that it can learn the alignment process using various types of network structures. As can be seen in this figure, we can confirm that DDDQN has slightly better performance than the faster DDDQN. However, in the complexity results in Table I, it was confirmed that the execution time of faster DDDQN is about two times faster than DDDQN. Based on these results, we were able to check the pros and cons of the proposed alignment networks. We also used the optimal window size up to 100 for each network in the next section to treat the actual sequence data.

**TABLE I:** Average Time Spent in Simulation For Various Networks (s)

Window size	10	30	50	100
DDDQN	1.916	2.910	2.522	2.911
faster DDDQN	1.080	1.327	1.302	1.411
Optimal alignment	143.6			

### C. Comparison With Conventional Alignment Algorithms

To confirm the difference between the DQNalign method and the conventional sequence alignment algorithm, three sequence sets were used: 1. HEV genome sequence set 2. two *E.coli* genome sequences 3. three mammalian genome sequences. In this section, we compared the exact match performance and time complexity between various sequence alignment methods: Clustal Omega, MUMmer, and the DQNalign method. The used detailed parameters are described in Table S6 and Table S7. Then, we implemented the pairwise alignment software based on the Clustal Omega from https:// github. com/etetoolkit/ext_apps/blob/master/src/clustal-omega-1.2.1. The implemented Python code is given in our Github link.

#### 1) Simulations on the HEV Genome Sequence Set

**Fig. 5.**
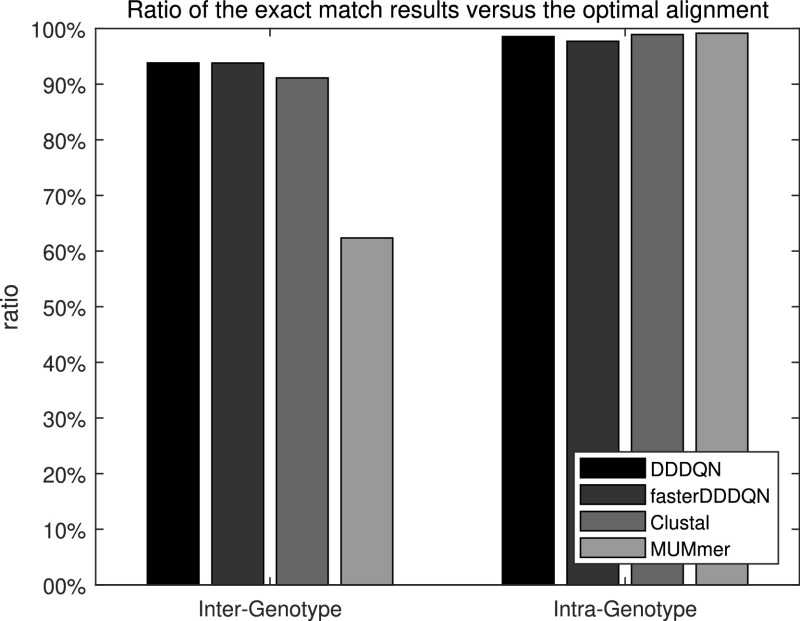
Exact match results in real sequences, which consist of 47 Hepatitis E Viruses. In this sequence set, there are 1081 sequence pairs.

The results of the HEV sequence set in Table S4 are shown in Fig. 5. All the alignment results are attached to the supplementary material S3. The ratio of exact matches in the heuristic alignment methods against optimal alignment was used for performance measure. As shown in Fig. 5, we could confirm that DQNalign and conventional methods show similar results to the optimal alignment for intra-genotype sequence pair cases, which have high identity values of above 0.8.

**Fig. 6.**
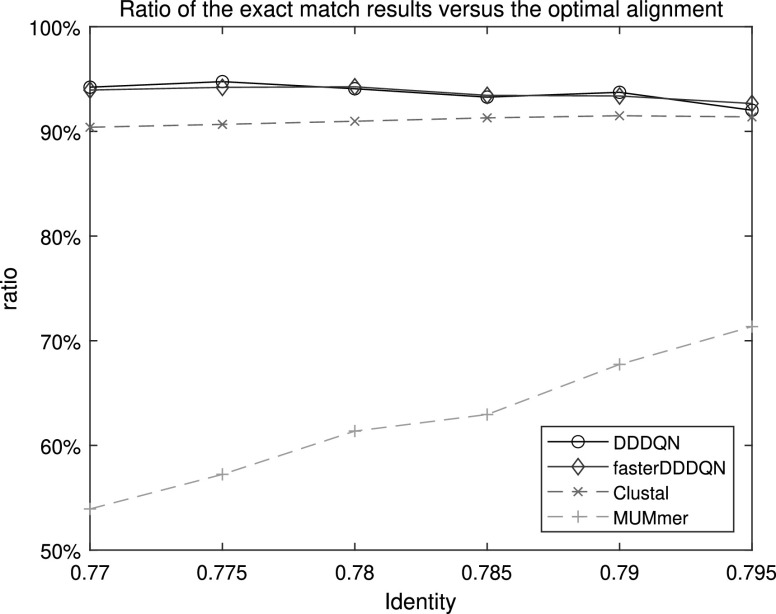
Exact match results in the case of inter-genotype, totally 749 cases of the sequence pairs are contained in this case.

However, in Fig. 6, we can observe that the difference between the alignment methods began to increase in the case of inter-genotype. In these inter-genotype cases, which occupy 69.3% of the entire sequence pairs, DDDQN and faster DDDQN show noticeably higher performance than the conventional alignment methods. The main reason for this difference is that in the case of dissimilar sequence pairs, the rapid decrease of the number of anchors (Ex. k-tuples in the Clustal method and MUMs in the MUMmer) causes the failure in the process connecting the anchors. Therefore, the conventional alignment methods could not complete the entire alignment and showed low coverage and exact matches. However, DQNalign method immediately estimated the window and decided the direction of alignment regardless of the anchors. Hence, the proposed DQNalign method has less difficulty in aligning the sequence pairs even in case of relatively low identity values.

#### 2) Simulations on the E.coli Genome Sequence Set

To observe the possibility of the alignment in long sequences, we did simulations on *Escherichia coli* O157 and *Escherichia coli* K-12. These alignment results are contained in Supplementary material S3. The brief alignment results are shown in Table II.

**TABLE II:** Alignment Results of the *E.coli* Sequences: Escherichia coliO157:H7 str. TW14359 vs. Escherichia Coli str. K-12 Substr. MG1655

	DQNalign	MUMmer	DQNalign with MUMmer	Clustal Omega
Exact match	2 359 437	3 990 344	4 132 349	3 890 193
Consumed time (s)	35 133	7.13	699	514 139

As shown in Table II, the proposed DQNalign has a lower exact match score compared to the other conventional methods owing to the insufficient pre-processing. As expected, sequence alignment for *E.coli* was not easy in case of using our DQNalign method. There are gene-scale genomic variations in the *E.coli* samples; therefore, it was hard to connect the whole sequences using only a few hundred-sized windows. Thus, we decided to additionally use the MUMmer method. Here, the overall improved genome alignment result could be obtained by combining MUMmer with our DQNalign method. We used the DQNalign method to align unaligned gaps of the alignment results obtained using the MUMmer methods. Using the DQNalign with MUMmer, we could properly align the entire *E.coli* sequences. And we can confirm that the DQNalign with MUMmer method achieves the best exact match performance than other conventional methods (MUMmer and Clustal).

In view of computational complexity, DQNalign method could align the entire sequence within a few hours. Also, DQNalign with MUMmer improves the exact matches in shorter time by providing an accurate preprocessing process (MUMmer) than DQNalign method. Moreover, we can confirmed that the proposed DQNalign method is much faster than the pairwise alignment algorithm of the Clustal Omega. However, we confirm that the MUMmer has a vastly high speed compared to other methods because the MUMmer method focuses on speed optimization in alignment between close sequence pairs.

#### 3) Simulations on the Mammalian Genome Sequence Set

Finally, we evaluated the performance of proposed DQNalign method for three most famous mammalian genome sequences: *Homo sapiens*, *Mus musculus*, and *Rattus norvegicus*. Since the proposed DQNalign method is one of global alignment methods, we simulated the performance of proposed and conventional methods in view of aligning the orthologous genes of these three organisms. We align the sequence pairs for the coding sequences of the genes of BRCA1, ELK1, and CCDC91, which are related to cancer incidence [25]–[27]. All sequence data were obtained by referring to NCBI's Gene search, and the sequence information is attached to Table S5.

As shown in Table III, the proposed DQNalign method showed the best performance in most sequence pairs. In the case of Mouse-Rat, we could confirm that the proposed DQNalign method has a similar performance to conventional methods. This is because the proposed DQNalign is specialized for sequence pairs with low similarities by training the networks in extreme environments, such as Human-Mouse and Rat-Human cases. Thus, the alignment results in Human-Mouse and Rat-Human showed a significantly higher alignment result than MUMmer and Clustal, which was similar to the inter-genotype case in HEV genomes. As expected, the proposed DQNalign showed higher alignment results than conventional methods in more extreme environments like the inter-genotype case in HEV genomes. Through these results, we can confirm that the proposed method performed better than conventional global alignment methods, even in mammalian gene analysis.

**TABLE III:** Alignment Results for the Mammalian Genomes

Case I	BRCA1		
	Human-Mouse	Mouse-Rat	Rat-Human
DQNalign	**3399**	4811	**3551**
Mummer	2273	**4821**	2771
Clustal	2456	4718	2482
Case II	ELK1		
	Human-Mouse	Mouse-Rat	Rat-Human
DQNalign	**1095**	**1237**	**1075**
Mummer	1018	**1237**	1002
Clustal	1051	1230	1041
Case III	CCDC91		
	Human-Mouse	Mouse-Rat	Rat-Human
DQNalign	**1034**	**1249**	**1021**
Mummer	826	**1249**	811
Clustal	979	1244	970

## IV. Discussion and Conclusion

In this paper, we proposed deep reinforcement learning based alignment method to reduce the complexity of pairwise alignment. Using only simple preprocessing based on the longest common substring (LCS), the DQNalign can align the entire sequences by repeating small alignments. We have confirmed that the DQNalign can achieve high accuracy and low complexity by adjusting the window size adaptively through the analysis.

We confirmed the advantages and disadvantages of the DQNalign method by analyzing the generated sequences and real HEV sequences. We observed that the DQNalign method has more accurate alignment performance than the conventional methods in dissimilar sequence pairs. We confirmed that the MUMmer method has a much faster speed than our DQNalign method. It is desirable to choose the DQNalign or the conventional alignment method in the consideration of real-time processing and accuracy.

The proposed DQNalign method showed the possibility of aligning two long sequences by combining conventional alignment algorithms. It was revealed that the sequences with millions of bases, such as E. coli, could be aligned by combining the DQNalign methods with the Clustal or the MUMmer. Through simulation results, we confirmed that the DQNalign method could be combined efficiently with conventional MUMmer and Clustal methods in view of improving accuracy.

Besides, we tried to find the proposed method's clinical significance by aligning the coding sequence of three type orthologous genes of Mammalian genome: *Homo sapiens*, *Mus musculus*, and *Rattus norvegicus*. DQNalign showed higher performance than conventional alignment methods in aligning genetically disease-intuitive genes such as BRCA1, ELK1, and CCDC91. Through these results, we could confirm that the proposed method is superior to the conventional global alignment in view of the actual clinics determining the presence or absence of disease through genetic testing or finding a biomarker.

In the future, we will attempt to apply the DQNalign to local alignment and multiple sequence alignment using improved learning methods and proper training strategies for each case. The DQNalign method used deep reinforcement learning based selection rather than human-based features; therefore, the alignment result was not stable for some sequence pairs. Hence, we will improve the stability of the DQNalign method using the alignment results of the several agents that are differently converged by deep reinforcement learning method. Besides, there were several challenges in securing performance on actual sequences, which result from the gap between the modeled sequences and the real sequences used in the learning. Therefore, we are going to solve these issues using a learning method that directly reflects the actual sequences.

## Supplementary Materials

Threesupplementary materials are attached to the submitted manuscript. First, we offer the detailed process of the DQNalign in Supplementary material S1. Then, the additional figures and tables are listed in the Supplementary material S2. Finally, the alignment results of the DQNalign are attached to the Supplementary material S3.

Three supplementary materials are attached to the submitted manuscript. First, we offer the detailed process of the DQNalign in Supplementary material S1. Then, the additional figures and tables are listed in the Supplementary material S2. Finally, the alignment results of the DQNalign are attached to the Supplementary material S3.
