# Supplementary material for: Pairwise Heuristic Sequence Alignment Algorithm Based on Deep Reinforcement Learning
Source: IEEE Open J Eng Med Biol. 2021 Jan 29;2:36–43. doi: 10.1109/OJEMB.2021.3055424 (PMC8901008; doi:10.1109/OJEMB.2021.3055424)
Supplement: Three supplementary materials are attached to the submitted manuscript. First, we offer the detailed process of the DQNalign in Supplementary material S1. Then, the additional figures and tables are listed in the Supplementary material S2. Finally, the alignment results of the DQNalign are attached  [file ojemb-3055424-mm.zip › OJEMB-3055424-MM/10022021082020_DQNalignOJEMB20210126SupplementaryS1.pdf]

Supplementary Materials

Pairwise heuristic sequence alignment algorithm based on deep reinforcement learning

Yong-Joon Song, Dong Jin Ji, Hyein Seo, Gyu Bum Han, and Dong-Ho Cho*, *Senior Member, IEEE*

THIS Supplementary Material deals with more details about the numerical proofs, network structure descriptions that were not mentioned in the manuscript of this paper. We hope you could fully understand about our proposed DQNalign method by this document.

I. Detailed numerical analysis on local best path selection model

In this section, we will provide detailed numerical analysis of the error probability of the alignment according to the change of window sizes. To analyze the error probability, problem, three assumptions, and basis of the numerical analysis will be defined. Then, we will prove the convergence of the error probability in case of a large window size.

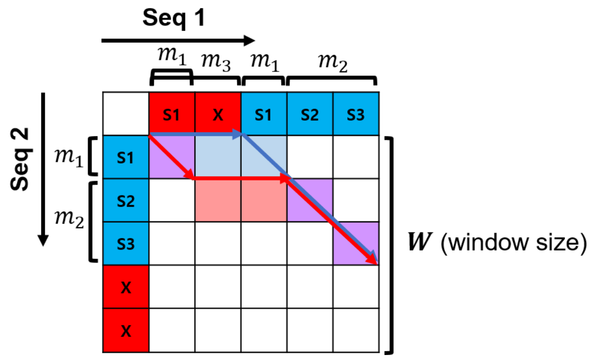

Fig. A1. Conceptual diagram of local best path selection model for analysis of error probability in case of fixed window size

A. Problem definition

In this paper, it is necessary to prove that our proposed method approached the optimal value as the window size increased, which is enabled by calculating the error probability at each step. According to previous reports, the probability distribution of the local and global alignment score follows the Gumbel distribution for two sequences with length n and m [14], [15], [16]. Therefore, we derived the equation that corresponds to the distribution of the alignment score and demonstrated how the increase of window size can improve the accuracy of the entire sequence alignment. As we mentioned in the main manuscript, we made three major assumptions and one constraint. First, we assume that the NW algorithm can precisely match the mutation information of the actual sequences. Second, it is assumed that several alignment paths are well-aligned when they have the same highest score in the current window. Third, the alignment at each step in the local best path selection method can be calculated independently. Conversely, for the constraint, we consider the alignment scoring parameters to prevent the indel preferences. Through these assumptions and constraint, we can derive the error probability in case of similar sequences that have positive alignment scores.

We want to explain why the DQNalign skips sub-alignment when the two sequences have the same subsequences in the start of the window, as shown in Fig.1a. Let us consider the case where indel occurred in sequence 1, as shown in Fig.A1. In this case, this indel sequence is identical to m_1 of the original sequence at the beginning. Here, the "red $S1$" and "blue $S1$" have the same nucleotides with length m_1. Next, m_2 denotes the length of the rest of the identical subsequences (blue subsequences), m_3 means the length of the indel (red subsequences), and the notation, X denotes "don't care" region.

Let us focus on the two paths shown in this figure. The blue path denotes the ground-truth alignment of the sequence pair, and the red path denotes the result of the sequence alignment obtained by our local best path selection method. Two sequence alignments have the same score, $score_{match} \times (m_1+m_2)+score_{gap} \times m_3$. Based on the second assumption, we can regard the red path as the correct path. Also, if we separate these two paths into two segments, the first segment can be seen as a small sequence alignment in the block with $m_1 \times (m_1+m_3)$ subsequences, and the shared remaining paths, $m_2 \times m_2$, are treated as the second segment.

We could demonstrate that these two paths have the same alignment score even if we consider only the first segment. In the first segment, the best score can be represented as $m_1 score_{match}+m_3 score_{gap}$. This value is equal to the red path score; therefore, we could state that the red path is also the best path. Therefore, we can infer that the forward alignment offers the best solution when the two sequences are equal at the beginning of the sequences regardless of the indel state. Hence, the proposed DQNalign skips the alignment process when the two sequences have the same subsequences in the start of the window. The detailed algorithm is depicted in Fig.1a.

B. Numerical analysis

When an error occurs in the local best path selection method with a fixed window size, we can narrow down the number of cases into two cases, as shown in Fig.2(a) and (b). In Fig.2(a), where SNP occurs at the first base pair in the window, the forward direction must be selected. However, when the local best path selection method takes a wrong insertion or deletion direction, it is assumed that an error occurs. In this case, let us consider the Gumbel distribution. All the notations of the equations are mentioned in the main article. The error probability that the score of the insertion or deletion path is larger than the actual score can be obtained as follows.

$$P(S(W,W-1)+score_{gap} > score_{answer}) \simeq 1-\exp(-KW(W-1)e^{-\lambda(score_{answer}-score_{gap})}) \quad (1)$$

Also, the total error probability $P_{e,SNP}$ that any one of the two cases will occur can be calculated as follows.

$$P_{e,SNP} \simeq 1-\exp(-KW(W-1)e^{-\lambda(score_{answer}-score_{gap})})^2 = 1-\exp(-2KW(W-1)e^{-\lambda(score_{answer}-score_{gap})}) \quad (2)$$

For a very large W, the above equation can be written as follows.

$$P_{e,SNP} \simeq \lim_{W\to\infty} 1-\exp(-2KW(W-1)e^{-\lambda(score_{answer}-score_{gap})}) \simeq 1-\exp(-2Ke^{\lambda score_{gap}} \lim_{W\to\infty} \frac{W^2}{e^{\lambda W score_{avg}}}) \quad (3)$$

When the average score per nucleotide is $score_{avg}$ and if it is greater than 0, we can see that $\lim_{W\to\infty} \frac{W^2}{e^{\lambda W score_{avg}}} \to 0$. Then, Eq.3 can be described approximately as follows.

$$P_{e,SNP} \simeq \lim_{W\to\infty} 1-\exp(-2Ke^{\lambda score_{gap}} \frac{W^2}{e^{\lambda W score_{avg}}}) \simeq \lim_{W\to\infty} 1-(1-2Ke^{\lambda score_{gap}} \frac{W^2}{e^{\lambda W score_{avg}}}) = \lim_{W\to\infty} 2Ke^{\lambda score_{gap}} \frac{W^2}{e^{\lambda W score_{avg}}} \to 0 \quad (4)$$

Using a similar method used in the derivation of Eq.1 to Eq.4, we can derive the error equations for the indel case. For the indel case, all of the cases that contain match, mismatch, and the other side indel are treated as errors. Then, we can derive the probability of each case expressed by the Gumbel distribution as follows.

$$\begin{aligned} &P(S(W,W-1)+score_{gap} > score_{ans}) \\ &\simeq 1-\exp(-KW(W-1)e^{-\lambda(score_{ans}-score_{gap})}) && \text{for indel} \\ &\simeq 1-\exp(-K(W-1)^2e^{-\lambda(score_{ans}-score_{match})}) && \text{for match} \\ &\simeq 1-\exp(-K(W-1)^2e^{-\lambda(score_{ans}-score_{mismatch})}) && \text{for mismatch} \end{aligned} \quad (5)$$

Here, we assume the probabilities of a match and mismatch to be 1:3, then the total error rate $P_{e,indel}$ is expressed as follows.

$$P_{e,indel} \simeq 1-\exp(-KW(W-1)e^{-\lambda(score_{ans}-score_{gap})}) \times (\frac{1}{4}\exp(-K(W-1)^2e^{-\lambda(score_{ans}-score_{match})}) + \frac{3}{4}\exp(-K(W-1)^2e^{-\lambda(score_{ans}-score_{mismatch})})) \quad (6)$$

For a very large W, the above equation can also be summarized as follows.

$$P_{e,indel} \simeq K(e^{\lambda score_{gap}} + \frac{1}{4}e^{\lambda score_{match}} + \frac{3}{4}e^{\lambda score_{mismatch}}) \times \lim_{W\to\infty} \frac{W^2}{e^{\lambda W score_{avg}}} \to 0 \quad (7)$$

TABLE A1. Parameter counts of various network architecture in DQNalign

Window size	DDDQN		faster DDDQN	
	Param.	FLOPS	Param.	FLOPS
10	763k	2.67M	103k	360k
30	1.68M	5.88M	74.0k	259k
50	2.60M	9.09M	107k	374k
100	4.70M	16.4M	172k	603k

When the rates of the SNP and indel are p_{SNP} and p_{indel}, respectively, the total error probability, $P_{e,total}$ can be expressed as follows.

$$P_{e,total} \simeq (p_{indel}K(e^{\lambda score_{gap}} + \frac{1}{4}e^{\lambda score_{match}} + \frac{3}{4}e^{\lambda score_{mismatch}}) + 2p_{SNP}Ke^{\lambda score_{gap}}) \times \lim_{W\to\infty} \frac{W^2}{e^{\lambda W score_{avg}}} \to 0 \quad (8)$$

II. Detailed Network Architecture

The detail of the deep Q-network based agent is shown in Fig.S1 and Fig.S2. Various techniques of the deep reinforcement learning are used for stability and performance. The detailed techniques will be dealt in this section.

A. Dueling Double Deep Q-network (DDDQN)

First, we apply Dueling Deep Q-network [11] and Double Deep Q-network [12] methods to improve the convergence and stability of Deep Q-network (DQN). Dueling DQN is a method that divides the predicted reward (Q value) into a kind of average and variance, and each of them is called as "Value" and "Advantage", respectively. Using this method, the agent can learn the scores of the states and actions separately, which helps the convergence of the learning progress.

Moreover, Double Deep Q-network method uses a duplicated network, which is called as target network. This target network is used for updating the main network while evading the overestimation. To solve the overestimation problem, Double DQN method uses the target network that converges slowly with preventing the policy from falling into the local minimum. In detail, we make the target network slowly converge towards the main network by constant ratio, tau.

Using these techniques, we define a Dueling Double Deep Q-network as shown in Fig.S1a. Variable window sizes are used as parameter of the convolutional neural networks. Each of the type of nucleotide (A,C,G,T) was converted to a 3 x 3 pixel square with CMYK color. To separate the left, right, top, and bottom end of the sub-sequences, 3 x 3 pixels of empty space are added. The detailed parameters of the network are shown in Fig.S1b. We can see that the number of parameters and FLOPS were linearly proportional to the window size as shown in Table A1. Additionally, we also use the experience replay methods to prevent the overestimation.

B. Faster Dueling Double Deep Q-network: separable convolutional layer based acceleration (faster DDDQN)

The DQNalign method is not limited to specific network architecture. We try to propose a second version of the network structure focused on reducing complexity. To reduce the complexity, the convolutional layer of the DDDQN is replaced with a separable convolutional layer. We call this modified version of the DDDQN as faster DDDQN. Separable convolutional layer separates a convolutional layer into two different layers called point-wise layer and depth-wise [13]. This method reduces the number of calculations required for the entire convolutional layer to $1/9$ times in case of 3 x 3 filters.

The modified version of DDDQN, faster DDDQN is shown in Fig.S2a. Like DDDQN, ACGT are mapped into CMYK, and an empty space is applied to the edge. However, in case of faster DDDQN, each nucleotide is mapped to a smaller size of 2 x 2 pixels. Then, we increase the number of layers to 4, unlike the DDDQN. The size of all filters is 3 x 3, and the size of stride is 3, 1, 1, and 3. Then, two maxpooling layers are added to reduce the size of the layer. Detailed parameters are noted in Fig.S2b.

As shown in Table A1, we can reduce the number of the operations from $1/9$ to $1/26$ times compared to the DDDQN using separable convolutional layer despite the increased numbers of the layers. Based on these results, we have confirmed that we can make adjustments between complexity and accuracy by controlling the architecture of the neural networks.

We can find the number of the parameters and FLOPS that have an abnormal tendency when the window size is 10. This behavior occurs owing to insufficient size of faster DDDQN, which makes it impossible to add the last convolutional layer. In this case, the faster DDDQN has only three convolutional layers instead of four. Absence of the last convolutional layer causes a large amount of increase in parameters and FLOPS in the fully connected layer. Thus, we can see that the numbers of parameters and FLOPS are large when the window size is 10 in Table A1.
