# Supplementary material for: Pairwise Heuristic Sequence Alignment Algorithm Based on Deep Reinforcement Learning
Source: IEEE Open J Eng Med Biol. 2021 Jan 29;2:36–43. doi: 10.1109/OJEMB.2021.3055424 (PMC8901008; doi:10.1109/OJEMB.2021.3055424)
Supplement: Three supplementary materials are attached to the submitted manuscript. First, we offer the detailed process of the DQNalign in Supplementary material S1. Then, the additional figures and tables are listed in the Supplementary material S2. Finally, the alignment results of the DQNalign are attached  [file ojemb-3055424-mm.zip › OJEMB-3055424-MM/10022021082020_DQNalignOJEMB20210126SupplementaryS2.pdf]

a)

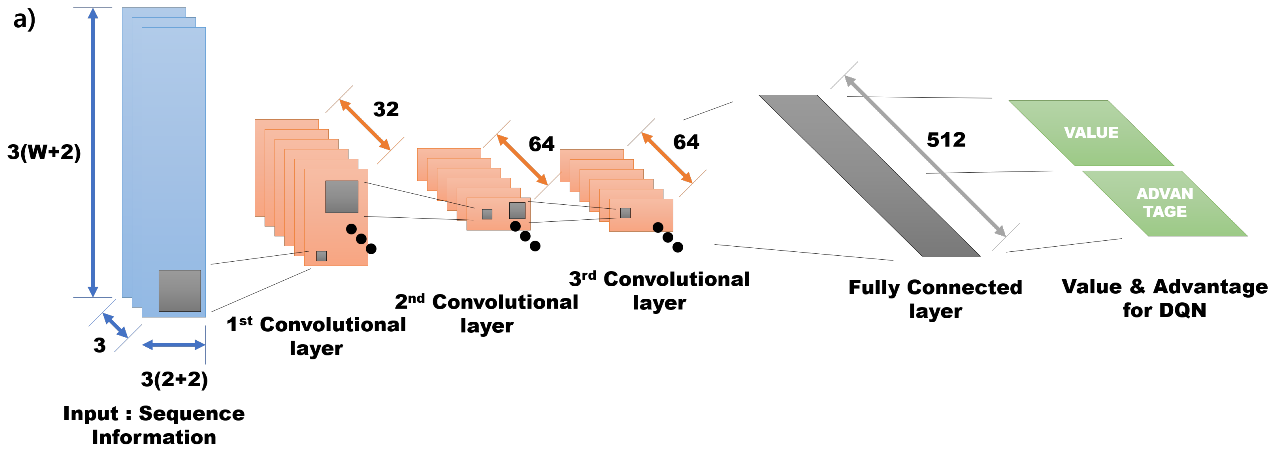

b)

Layer type	Conv	Conv	Conv	FC	split	FC / FC (Value / Advan.)
Stride	3 x 3	3 x 3	1 x 1	1 x 1	-	1 x 1 / 1 x 1
Padding	Same	Same	Same	Valid	-	Valid
Filter Shape	9 x 9 x 32	6 x 6 x 64	3 x 3 x 64	⌈(W+2)/3⌉ x 2 x 64 x 512	-	256 x 1 / 256 x 3
Input size	3(W+2) x 12 x 3	(W+2) x 4 x 32	⌈(W+2)/3⌉ x 2 x 64	⌈(W+2)/3⌉ x 2 x 64	512	256 / 256
Output size	(W+2) x 4 x 32	⌈(W+2)/3⌉ x 2 x 64	⌈(W+2)/3⌉ x 2 x 64	512	256 / 256	1 / 3

Fig. S1. Detailed network architecture of Dueling Double Deep Q network (DDDQN). a) Conceptual diagram of network. b) Parameter table of network

a)

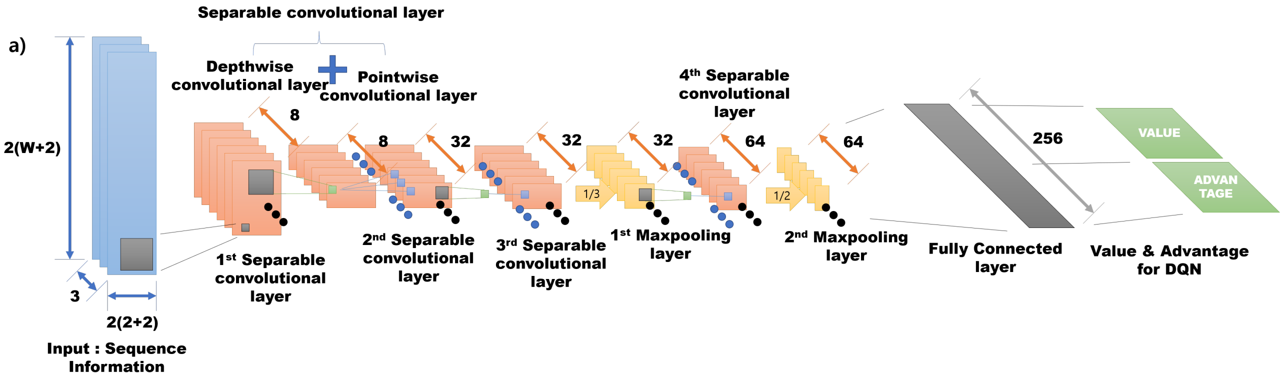

b)

Layer type	Conv dw	Conv pw	Conv dw	Conv pw	Conv dw	Conv pw	Maxpool
Stride	3 x 3	-	1 x 1	-	1 x 1	-	3 x 1
Padding	Same	Same	Same	Same	Same	Same	Valid
Filter Shape	3 x 3 x 3 dw	1 x 1 x 3 x 8	3 x 3 x 3 dw	1 x 1 x 8 x 32	3 x 3 x 32 dw	1 x 1 x 32 x 32	Pool 3 x 1
Input size	2(W+2) x 8 x 3	⌈2(W+2)/3⌉ x 3 x 3	⌈2(W+2)/3⌉ x 3 x 8	⌈2(W+2)/3⌉ x 3 x 8	⌈2(W+2)/3⌉ x 3 x 32	⌈2(W+2)/3⌉ x 3 x 32	⌈⌈2(W+2)/3⌉/3⌉ x 3 x 32
Output size	⌈2(W+2)/3⌉ x 3 x 3	⌈2(W+2)/3⌉ x 3 x 8	⌈2(W+2)/3⌉ x 3 x 8	⌈2(W+2)/3⌉ x 3 x 32	⌈2(W+2)/3⌉ x 3 x 32	⌈2(W+2)/3⌉ x 3 x 32	⌈⌈2(W+2)/3⌉/3⌉ x 3 x 32

Continued

Conv dw	Conv pw	Maxpool	FC	split	FC / FC (Value / Advan.)
3 x 3	-	2 x 1	-	-	- / -
Same	Same	Valid	Valid	-	Valid / Valid
3 x 3 x 32 dw	1 x 1 x 32 x 64	Pool 2 x 1	⌈⌈⌈2(W+2)/3⌉/3⌉/3⌉/2⌉ x 1 x 64 x 256	-	128 x 1 / 128 x 3
⌈⌈2(W+2)/3⌉/3⌉ x 3 x 32	⌈⌈⌈2(W+2)/3⌉/3⌉/3⌉ x 1 x 32	⌈⌈⌈2(W+2)/3⌉/3⌉/3⌉ x 1 x 64	⌈⌈⌈⌈2(W+2)/3⌉/3⌉/3⌉/2⌉ x 1 x 64	256	128 / 128
⌈⌈⌈2(W+2)/3⌉/3⌉/3⌉ x 1 x 32	⌈⌈⌈2(W+2)/3⌉/3⌉/3⌉ x 1 x 64	⌈⌈⌈⌈2(W+2)/3⌉/3⌉/3⌉/2⌉ x 1 x 64	256	128 / 128	1 / 3

Fig. S2. Detailed network architecture of separable convolutional layer based network (faster DDDQN). a) Conceptual diagram of network. b) Parameter table of network

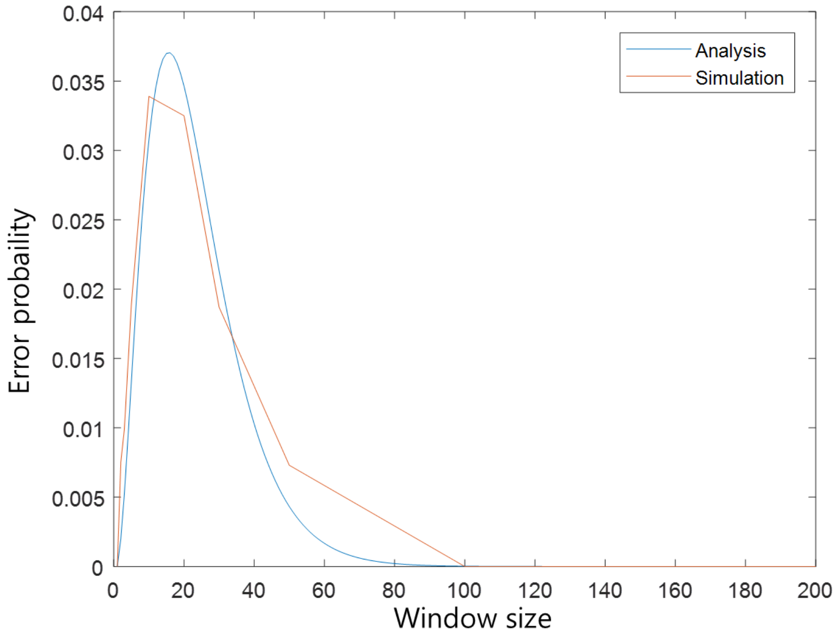

Fig. S3. Numerical analysis and simulation results for window size and step error probability

TABLE S1. Detailed parameters of sequence generation for training procedure

Parameters	Training environment
Sequence length (l)	8000
Probability of SNP (p_{SNP})	0.1
Probability of indel (p_{indel})	0.02
Maximum length of indel (I_{max})	10
Zipfian distribution parameter (s)	1.6

TABLE S2. Detailed parameters for validation of numerical analysis

Parameters	Value
Sequence length (l)	8000
Probability of SNP (p_{SNP})	0.067
Probability of indel (p_{indel})	0.00014
Maximum length of indel (I_{max})	10
Zipfian distribution parameter (s)	1.6

TABLE S3. Detailed simulation environment

Parameters	Case 1<br>Only SNP	Case 2-1<br>Only indel (I_{max})	Case 2-2<br>Only indel (p_{indel})	Case 3<br>SNP&indel	Case 4<br>independent
Sequence length (l)	8000	8000	8000	8000	8000
Probability of SNP (p_{SNP})	0.05,0.1,0.2	0	0	0.1	1
Probability of indel (p_{indel})	0	0.1	0.05,0.1,0.2	0.1	0
Maximum length of indel (I_{max})	0	1,2,3	2	1,2,3	0
Zipfian distribution parameter (s)	1.6	1.6	1.6	1.6	1.6

TABLE S4. Benchmarked HEV genome sequences

NO.	STRAIN NAME	ACCESSION NO.	GENOTYPE	LENGTH
1	B1	M73218	I	7207
2	B2	D10330	I	7194
3	I3	AF076239	I	7194
4	NP1	AF051830	I	7199
5	P2	AF185822	I	7143
6	Yam-67	AF459438	I	7206
7	C1	D11092	I	7207
8	C2	L25595	I	7221
9	C3	L08816	I	7176
10	C4	D11093	I	7194
11	China Hebei	M94177	I	7200
12	P1	M80581	I	7138
13	I1	X98292	I	7202
14	Morocco	AY230202	I	7212
15	T3	AY204877	I	7170
16	M1	M74506	II	7180
17	HE-JA10	AB089824	III	7262
18	JKN-Sap	AB074918	III	7256
19	JMY-HAW	AB074920	III	7240
20	SW-US1	AF082843	III	7207
21	US1	AF060668	III	7202
22	US2	AF060669	III	7277
23	JBOAR1-HYO04	AB189070	III	7247
24	JDEER-HYO03L	AB189071	III	7230
25	JJT-KAN	AB091394	III	7218
26	JIMO-HYO03L	AB189072	III	7180
27	JRA1	AP003430	III	7230
28	JSO-HYO03L	AB189073	III	7180
29	JTH-HYO03L	AB189074	III	7180
30	JYO-HYO03L	AB189075	III	7180
31	SWJ570	AB073912	III	7257
32	KYRGYZ	AF455784	III	7239
33	ARKELL	AY115488	III	7255
34	HE-JA1	AB097812	IV	7258
35	HE-JK4	AB099347	IV	7250
36	HE-JI4	AB080575	IV	7186
37	JAK-Sai	AB074915	IV	7236
38	JKK-SAP	AB074917	IV	7235
39	JSM-SAP94	AB161717	IV	7202
40	JSN-SAP-FH	AB091395	IV	7234
41	JSN-SAP-FH02C	AB200239	IV	7251
42	JTS-SAP02	AB161718	IV	7202
43	JYW-SAP02	AB161719	IV	7202
44	SWJ13-1	AB097811	IV	7258
45	SWCH25	AY594199	IV	7270
46	T1	AJ272108	IV	7232
47	CCC220	AB108537	IV	7193

TABLE S5. Benchmarked Gene ID in mammalian simulations

ORGANISM	REFERENCE GENOME VER.	GENE NAME	NCBI Gene ID	LENGTH of CDS
Homo sapiens	GRCh38.p13	BRCA1	672	5529
Homo sapiens	GRCh38.p13	ELK1	2002	1287
Homo sapiens	GRCh38.p13	CCDC91	55297	1326
Mus musculus	GRCm39	BRCA1	12189	5439
Mus musculus	GRCm39	ELK1	13712	1290
Mus musculus	GRCm39	CCDC91	67015	1329
Rattus norvegicus	Rnor_6.0	BRCA1	497672	5454
Rattus norvegicus	Rnor_6.0	ELK1	314436	1284
Rattus norvegicus	Rnor_6.0	CCDC91	312863	1329

TABLE S6. Parameters used in the Clustal Omega software

Parameters	HEV simulation case	E.coli simulation case	Mammalian simulation case
K (k-tuple)	2	7	2
signif (Number of top diagonals to select)	4	500	4
window (Allowable differences of diagonals nearby selected top diagonals)	4	5	4
wind_gap (Allowable gaps for merging top diagonals)	5	5	5

TABLE S7. Parameters used in the MUMmer software

Parameters	HEV simulation case	E.coli simulation case	Mammalian simulation case
max_gap (Maximum length of gaps which can be added into cluster)	90	100000	90
min_cluster (Minimum length of cluster)	20	10000	20
