# Supplementary material for: Pairwise Heuristic Sequence Alignment Algorithm Based on Deep Reinforcement Learning
Source: IEEE Open J Eng Med Biol. 2021 Jan 29;2:36–43. doi: 10.1109/OJEMB.2021.3055424 (PMC8901008; doi:10.1109/OJEMB.2021.3055424)
Supplement: Three supplementary materials are attached to the submitted manuscript. First, we offer the detailed process of the DQNalign in Supplementary material S1. Then, the additional figures and tables are listed in the Supplementary material S2. Finally, the alignment results of the DQNalign are attached  [file ojemb-3055424-mm.zip › OJEMB-3055424-MM/README.pdf]

DESCRIPTION: The supplementary files include information related to the main paper.

SIZE: 80.7 MB

PLAYER INFORMATION: N/A

PACKING LIST: OJEMB-3055424-MM.zip

CONTACT INFORMATION:
Prof. Dong-Ho Cho
dhcho@kaist.ac.kr
